# Supplementary material for: Environmental modulation of social isolation-induced sleep loss in Drosophila
Source: bioRxiv. 2026 Oct 2:2026.09.23.753895. Preprint. [Version 2] doi: 10.64898/2026.09.23.753895 (PMC13648406; doi:10.64898/2026.09.23.753895)
Supplement: Supplementary file 1 [file NIHPP2026.09.23.753895v2-supplement-2.pdf]

bioRxiv preprint doi: https://doi.org/10.64898/2026.09.23.753895; this version posted October 2, 2026. The copyright holder for this preprint (which was not certified by peer review) is the author/funder, who has granted bioRxiv a license to display the preprint in perpetuity. It is made available under a CC-BY 4.0 International license.

Supporting Information

Supplemental Figures S1-S6

Supplemental Video 1

bioRxiv preprint doi: https://doi.org/10.64898/2026.09.23.753895; this version posted October 2, 2026. The copyright holder for this preprint (which was not certified by peer review) is the author/funder, who has granted bioRxiv a license to display the preprint in perpetuity. It is made available under a CC-BY 4.0 International license.

Figure S1

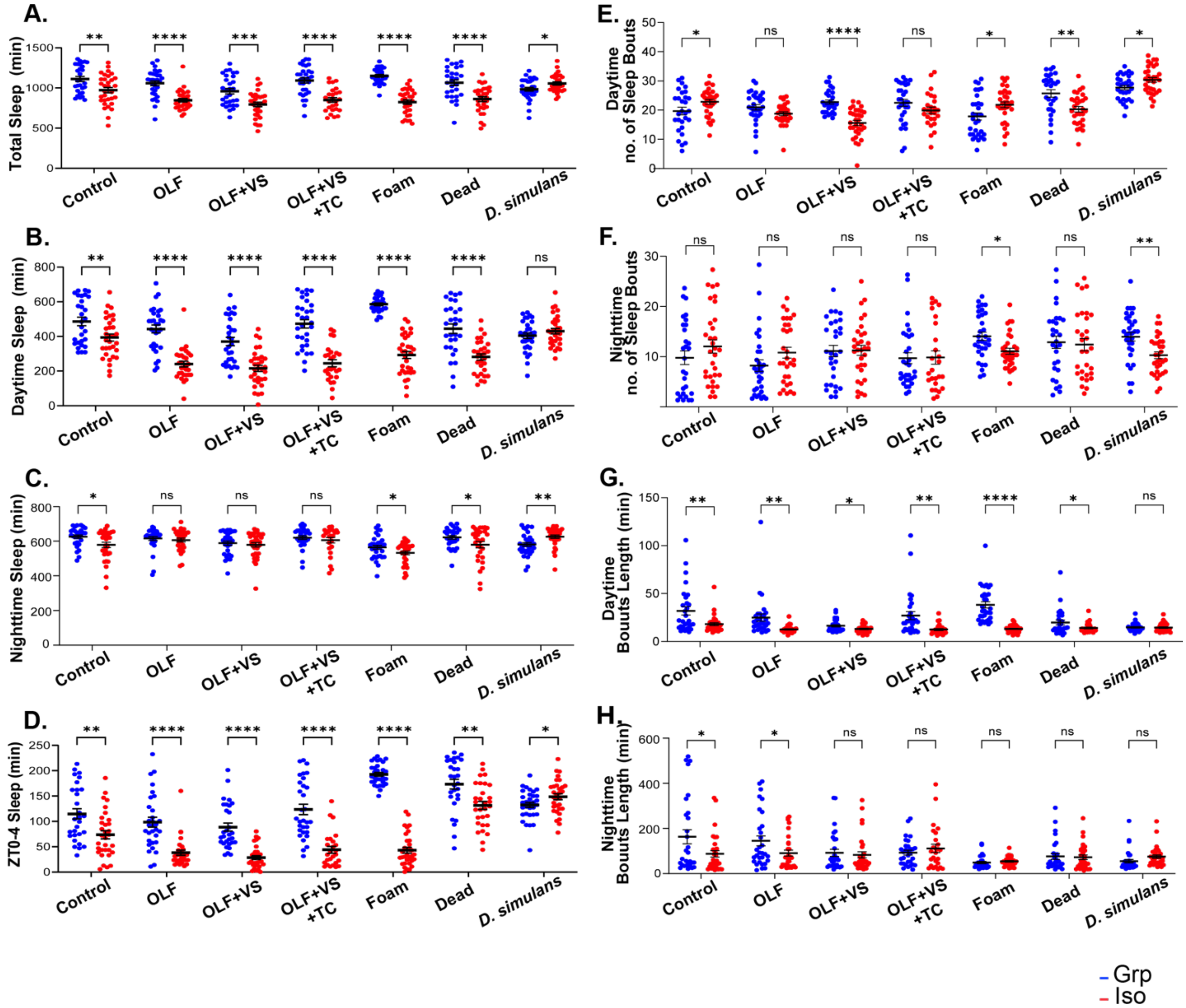

Figure S1. Sensory cues from conspecifics do not alleviate chronic social isolation-induced sleep loss. (A–D) Quantification of total sleep (A), daytime sleep (B), nighttime sleep (C), and ZT0-4 sleep (D). (E–H) Quantification of daytime sleep-bout number (E), nighttime sleep-bout number (F), mean daytime sleep-bout length (G), and mean nighttime sleep-bout length (H). Flies were maintained under the experimental conditions described in Figure 1: Control, OLF (olfactory cues), OLF+VS (olfactory and visual cues), OLF+VS+TC (olfactory, visual, and tactile cues), Foam, Dead conspecifics, and live *D. simulans*. Blue symbols denote grouped flies, and red symbols denote isolated flies. Each dot represents one fly; data are presented as mean ± s.e.m. Statistical analysis was performed using two-sided unpaired t-tests with Welch's correction. *P < 0.05; **P < 0.01; ***P < 0.001; ****P < 0.0001; NS, not significant; n = 29–32 flies per condition.

bioRxiv preprint doi: https://doi.org/10.64898/2026.09.23.753895; this version posted October 2, 2026. The copyright holder for this preprint (which was not certified by peer review) is the author/funder, who has granted bioRxiv a license to display the preprint in perpetuity. It is made available under a CC-BY 4.0 International license.

Figure S2:

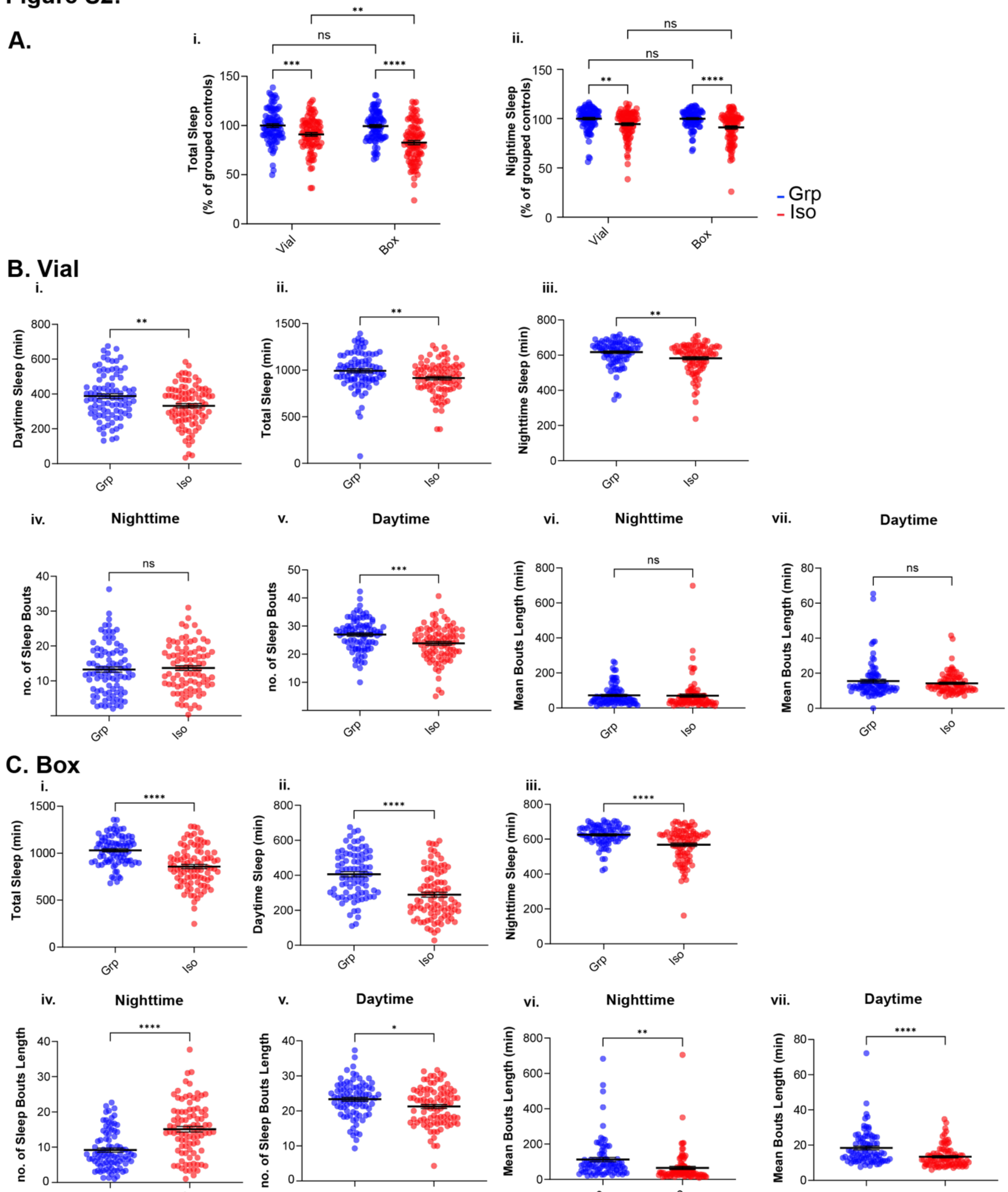

Figure S2. Spatial restriction exacerbates chronic social isolation-induced sleep loss (A) Quantification of total sleep and nighttime sleep expressed as a percentage of grouped controls, normalized to 100% (mean ± s.e.m.; individual data points shown). Statistical analysis was performed using two-way ANOVA followed by Fisher's LSD multiple-comparison tests. (B,C) Quantification of all sleep parameters of grouped and isolated animals using (B) vial-based and (C) box-based housing systems. Each dot represents one fly; data are presented as mean ± s.e.m. Statistical analysis was

bioRxiv preprint doi: https://doi.org/10.64898/2026.09.23.753895; this version posted October 2, 2026. The copyright holder for this preprint (which was not certified by peer review) is the author/funder, who has granted bioRxiv a license to display the preprint in perpetuity. It is made available under a CC-BY 4.0 International license.

performed using two-sided unpaired t-tests with Welch's correction. *$P < 0.05$; **$P < 0.01$; ***$P < 0.001$; ****$P < 0.0001$; NS, not significant; n = 86–92 flies per condition.

bioRxiv preprint doi: https://doi.org/10.64898/2026.09.23.753895; this version posted October 2, 2026. The copyright holder for this preprint (which was not certified by peer review) is the author/funder, who has granted bioRxiv a license to display the preprint in perpetuity. It is made available under a CC-BY 4.0 International license.

Figure S3

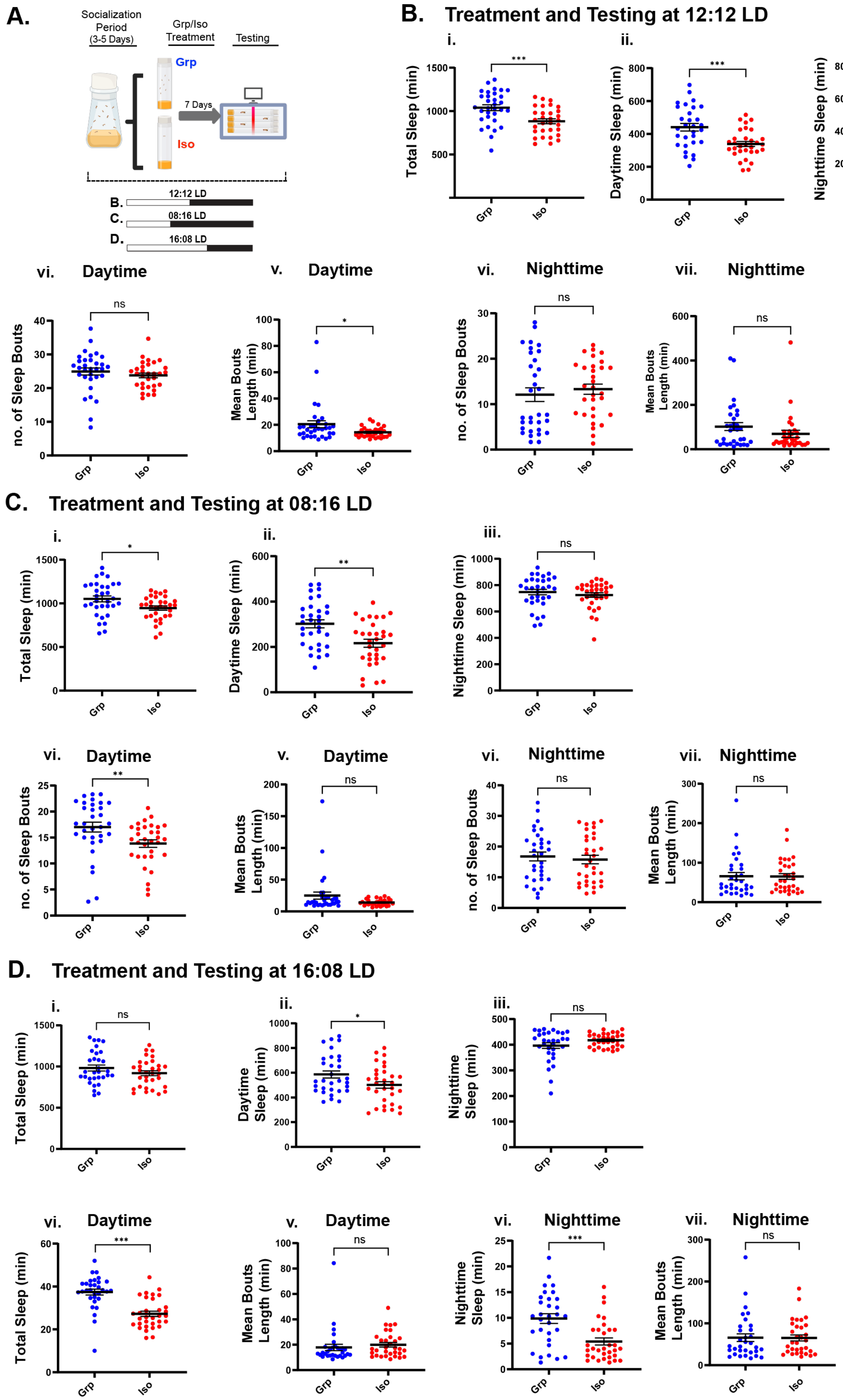

bioRxiv preprint doi: https://doi.org/10.64898/2026.09.23.753895; this version posted October 2, 2026. The copyright holder for this preprint (which was not certified by peer review) is the author/funder, who has granted bioRxiv a license to display the preprint in perpetuity. It is made available under a CC-BY 4.0 International license.

Figure S3. Chronic social isolation alters sleep under different seasonal photoperiods.
(A) Schematic of the experimental design. Flies were socialized for 3–5 days and then group-housed (Grp) or isolated (Iso) for 7 days under (B) 12:12 LD, (C) 08:16 LD, and (D) 16:08 LD photoperiods. Following treatment, locomotor activity was recorded under photoperiods matched to those used during treatment.
(B–D) Quantification of sleep parameters in grouped and chronically isolated flies under (B) equinox-like 12:12 LD (n = 31–32), (C) winter-like 08:16 LD (n = 32), and (D) summer-like 16:08 LD (n = 31–32) conditions. Data are presented as mean ± s.e.m.; individual data points are shown. Statistical analysis was performed using two-sided unpaired t-tests with Welch's correction. **P* < 0.05; ***P* < 0.01; ****P* < 0.001; *****P* < 0.0001; NS, not significant.

bioRxiv preprint doi: https://doi.org/10.64898/2026.09.23.753895; this version posted October 2, 2026. The copyright holder for this preprint (which was not certified by peer review) is the author/funder, who has granted bioRxiv a license to display the preprint in perpetuity. It is made available under a CC-BY 4.0 International license.

Figure S4

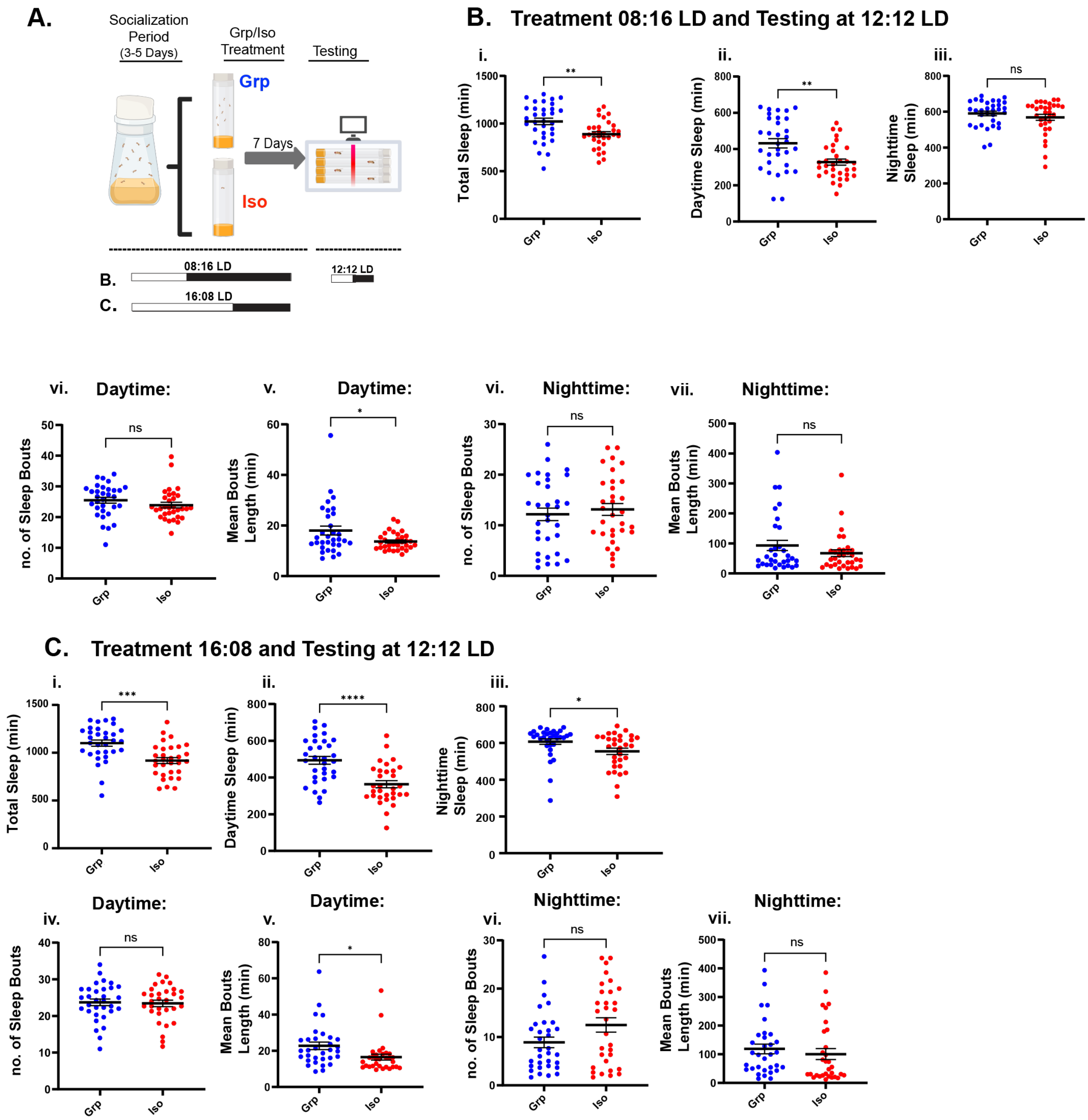

Figure S4. Chronic social isolation alters sleep under different seasonal photoperiods.

(A) Schematic of the experimental design. Flies were socialized for 3–5 days and then group-housed (Grp) or isolated (Iso) for 7 days under (B) 08:16 LD or (C) 16:08 LD photoperiods. Following treatment, locomotor activity was recorded under standard 12:12 LD conditions.

(B–D) Quantification of total, daytime, and nighttime sleep, as well as daytime and nighttime sleep-bout number and mean bout length, under each treatment photoperiod. Data are presented as mean ± s.e.m.; individual data points are shown. Statistical

bioRxiv preprint doi: https://doi.org/10.64898/2026.09.23.753895; this version posted October 2, 2026. The copyright holder for this preprint (which was not certified by peer review) is the author/funder, who has granted bioRxiv a license to display the preprint in perpetuity. It is made available under a CC-BY 4.0 International license.

comparisons were performed using two-sided unpaired t-tests with Welch's correction. *$P < 0.05$; **$P < 0.01$; ***$P < 0.001$; ****$P < 0.0001$; NS, not significant; n = 30–32 flies per condition.

bioRxiv preprint doi: https://doi.org/10.64898/2026.09.23.753895; this version posted October 2, 2026. The copyright holder for this preprint (which was not certified by peer review) is the author/funder, who has granted bioRxiv a license to display the preprint in perpetuity. It is made available under a CC-BY 4.0 International license.

Figure S5

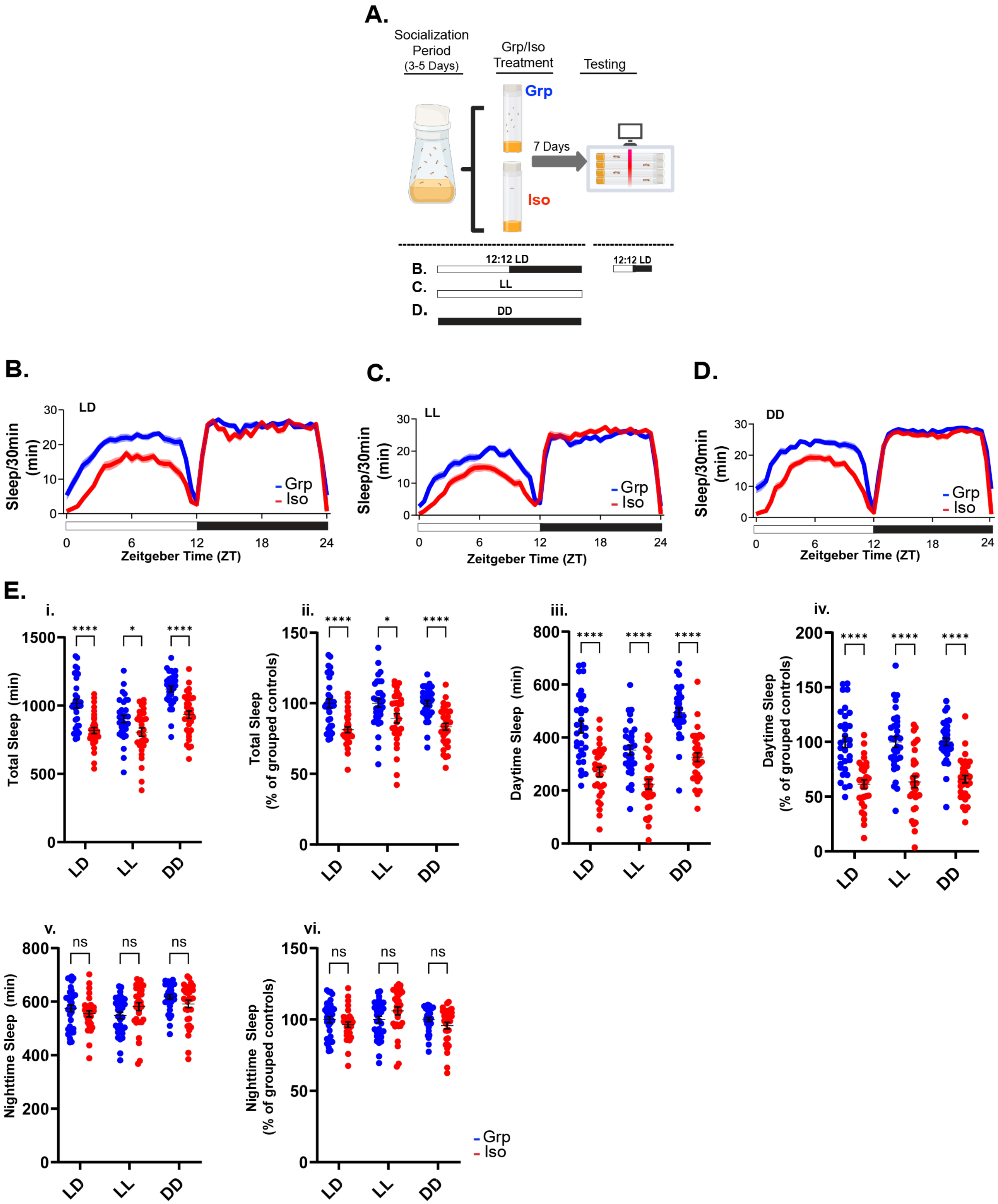

Figure S5. Chronic social isolation reduces sleep independently of environmental light conditions during treatment.

bioRxiv preprint doi: https://doi.org/10.64898/2026.09.23.753895; this version posted October 2, 2026. The copyright holder for this preprint (which was not certified by peer review) is the author/funder, who has granted bioRxiv a license to display the preprint in perpetuity. It is made available under a CC-BY 4.0 International license.

(A) Schematic of the experimental design.

(B-D) Sleep profiles of group-housed and isolated flies following 3–5 days of socialization and 7 days of group or isolation treatment under (B) 12:12 LD, (C) constant light (LL), or (D) constant darkness (DD), with behavioral testing performed under the 12:12 LD condition.

(E) Quantification of daytime, total, and nighttime sleep under each lighting condition, expressed as both a percentage of grouped controls and absolute sleep duration (min): (i–ii) daytime sleep, (iii–iv) total sleep, and (v–vi) nighttime sleep. Data are presented as mean ± s.e.m.; individual data points are shown. Statistical comparisons were performed using two-sided unpaired t-tests. *$P < 0.05$; **$P < 0.01$; ***$P < 0.001$; ****$P < 0.0001$; NS, not significant; n = 30–32 flies per group.

bioRxiv preprint doi: https://doi.org/10.64898/2026.09.23.753895; this version posted October 2, 2026. The copyright holder for this preprint (which was not certified by peer review) is the author/funder, who has granted bioRxiv a license to display the preprint in perpetuity. It is made available under a CC-BY 4.0 International license.

Figure S6

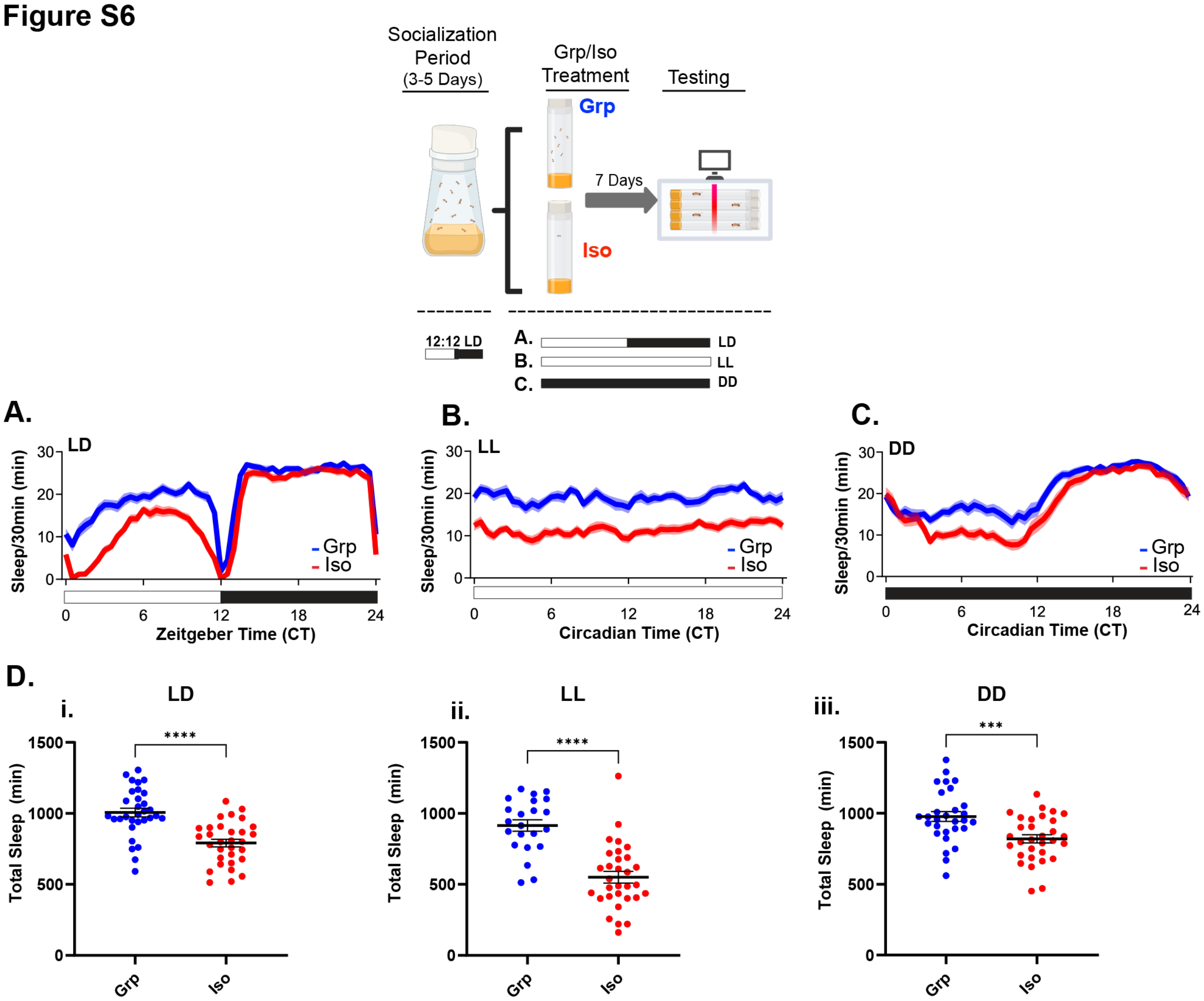

Figure S6. Chronic social isolation reduces sleep independently of environmental light conditions during treatment and testing.
(A–C) Schematic of the experimental design. Sleep profiles of group-housed and isolated flies following 3–5 days of socialization and 7 days of group or isolation treatment under (A) 12:12 LD, (B) constant light (LL), or (C) constant darkness (DD), with behavioral testing performed under the corresponding lighting condition.
(D) Isolated flies exhibited reduced total sleep under LD (****$P < 0.0001$), LL (****$P < 0.0001$), and DD (***$P < 0.001$). n = 29–31 flies per condition. Each dot represents one fly; data are presented as mean ± s.e.m. Statistical comparisons were performed using two-sided unpaired t-tests with Welch's correction.
